# Virus-like particles: preparation, immunogenicity and their roles as nanovaccines and drug nanocarriers

**DOI:** 10.1186/s12951-021-00806-7

**Published:** 2021-02-25

**Authors:** Saghi Nooraei, Howra Bahrulolum, Zakieh Sadat Hoseini, Camellia Katalani, Abbas Hajizade, Andrew J. Easton, Gholamreza Ahmadian

**Affiliations:** 1 Department of Industrial and Environmental Biotechnology, National Institute of Genetic Engineering and Biotechnology (NIGEB), P. O. BOX: 14155-6343, Tehran, 1497716316 Iran; 2 Sari Agriculture Science and Natural Resource University (SANRU), Genetics and Agricultural Biotechnology Institute of Tabarestan (GABIT), Sari, Iran; 3 Applied Microbiology Research Center, Systems Biology and Poisonings Institute, Baqiyatallah University of Medical Sciences, Tehran, Iran; 4 School of Life Sciences, Gibbet Hill Campus, University of Warwick, Coventry, UK

**Keywords:** Virus-like particles (VLPs), Subunit vaccine, Expression and purification platforms, Infectious disease vaccine, Cancer vaccine, Immune response

## Abstract

Virus-like particles (VLPs) are virus-derived structures made up of one or more different molecules with the ability to self-assemble, mimicking the form and size of a virus particle but lacking the genetic material so they are not capable of infecting the host cell. Expression and self-assembly of the viral structural proteins can take place in various living or cell-free expression systems after which the viral structures can be assembled and reconstructed. VLPs are gaining in popularity in the field of preventive medicine and to date, a wide range of VLP-based candidate vaccines have been developed for immunization against various infectious agents, the latest of which is the vaccine against SARS-CoV-2, the efficacy of which is being evaluated. VLPs are highly immunogenic and are able to elicit both the antibody- and cell-mediated immune responses by pathways different from those elicited by conventional inactivated viral vaccines. However, there are still many challenges to this surface display system that need to be addressed in the future. VLPs that are classified as subunit vaccines are subdivided into enveloped and non- enveloped subtypes both of which are discussed in this review article. VLPs have also recently received attention for their successful applications in targeted drug delivery and for use in gene therapy. The development of more effective and targeted forms of VLP by modification of the surface of the particles in such a way that they can be introduced into specific cells or tissues or increase their half-life in the host is likely to expand their use in the future. Recent advances in the production and fabrication of VLPs including the exploration of different types of expression systems for their development, as well as their applications as vaccines in the prevention of infectious diseases and cancers resulting from their interaction with, and mechanism of activation of, the humoral and cellular immune systems are discussed in this review. 
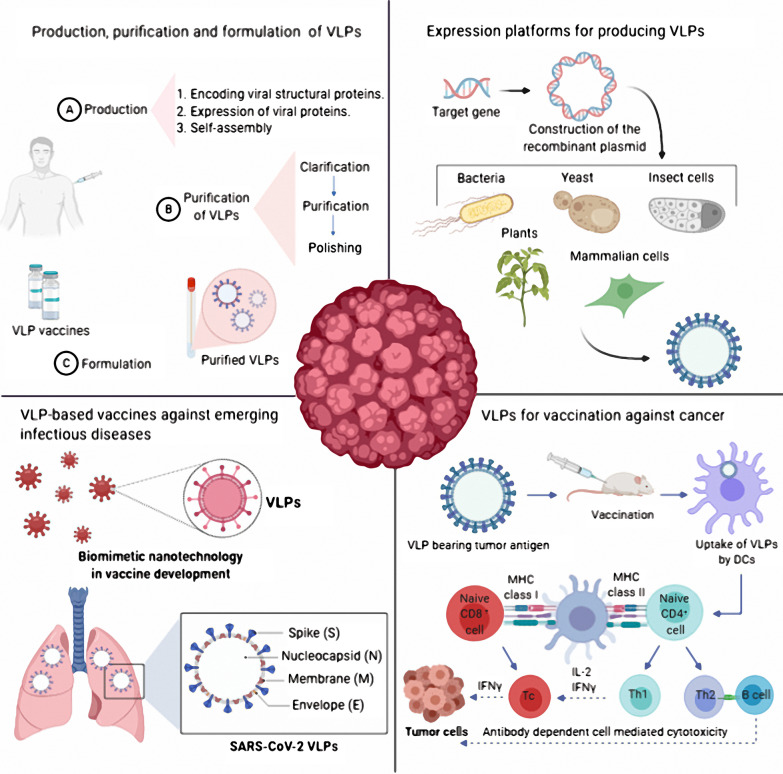

## Background

Viral-like particles (VLPs) are nanoscale structures made up of assembled viral proteins that lack viral genetic material and are therefore non-infectious [1]. VLPs are dispersed nanomaterials that can be produced in a variety of systems, including mammals, plants, insects, and bacteria. VLPs can be exploited as carriers for the delivery of bio- and nanomaterials, such as drugs, vaccines, quantum dots and imaging substances by virtue of the cavity within their structure [2, 3]. VLPs are icosahedral or rod-shaped structures made by the self-assembly of viral structural proteins [4]. These nanoparticle structures were first identified in 1968 in the sera of patients with Down's syndrome, leukemia and hepatitis. However, their biological nature remained unknown, though it was shown that there are antigenic sites on the surface of these particles [5]. Subsequently it was shown that virus capsid, envelope and, sometimes, core viral proteins can form VLP structures. VLPs can be experimentally generated in the laboratory using recombinant viral proteins that are expressed in a range of expression systems including prokaryotic cells [6], yeast [7], insect cell lines [8, 9], plants [10] and mammalian cell lines [11, 12]. While VLPs are commonly produced using proteins(s) from a single virus type, chimeric VLPs can also be created by the assembly of structural proteins from different viruses [6].

Structural proteins from viruses, such as human immunodeficiency virus (HIV), adeno-associated virus, Hepatitis B virus (HBV), Hepatitis C virus (HCV) and bacteriophages have been used to produce VLPs [9–11]. These particles are of different sizes, with most ranging from 20–200 nm. VLPs are highly organized structures and because of their underlying geometry, they resemble pathogen-associated structural patterns (PASP) that can be efficiently recognized by the cells and molecules of the immune system [12, 13]. Based on the presence or absence of lipid envelopes, VLPs are classified into two main types: enveloped and non-enveloped VLPs and the presence of proteins organized into single-layered, two-layered or multi-layered [14]. VLPs are being used for different purposes. Since they contain an internal cavity, they can be used as efficient delivery vehicles and they have been exploited for the delivery of different biological material, including genes, peptides, proteins and small drugs. An attractive feature is that they can be used for targeted drug delivery and their property of enhanced permeability and retention make these carriers an attractive means of drug delivery to tumor tissues for delivering treatment and also for tumor imaging [15–17].

A largely exploited application of VLPs is their potential in vaccinology where they can offer several advantages over conventional vaccine approaches [18–20]. Because of their size and shape, which resembles the actual size and shape of native viruses, these structures can efficiently elicit the immune responses and in VLPs lacking viral genomes there is no potential for replication within the target cells, which offers improved safety especially for immunocompromised or elderly vaccinees [21]. While VLPs can stimulate both humoral and cellular immune responses [22, 23] they can also be loaded with immune-modulators, such as innate immune system stimuli to provoke more effective immune responses. Several VLP-based vaccines have been approved for use in the clinic and are now commercially available with others in various phases of clinical trials (Table 1). This review article describes the classification of VLPs and considers the immunogenicity of VLP-based vaccines. Different expression systems for recombinant protein production and production of VLP proteins are discussed. Applications of VLPs as vaccines in the prevention of infectious diseases and cancers, as well as their future prospects, are discussed.

**Table 1:** FDA approved or clinical trial stage VLP-based vaccines against infectious diseases

Trade name	Infectious agent	Target disease	Status	Company	Antigen(s)	Expression System
Gardasil®	Human Papilloma Virus (HPV)	Human papillomavirus, Types 6, 11, 16, 18, 31, 33, 45, 52, and 58	Approved	Merck	Major capsid protein L1 epitope of HPV types 6, 11, 16, and 18	Yeast
Gardasil9®	Human Papilloma Virus (HPV)	Human papillomavirus Types 6, 11, 16, 18, 31, 33, 45, 52, and 58	Approved	Merck	Major capsid protein L1 epitope of HPV types 6, 11, 16, 18, 31, 33, 45, 52, and 58	Yeast
Cervarix®	Human Papilloma Virus (HPV)	Human papillomavirus (Types 16 and 18)	Approved	Glaxo-SmithKline Inc	Major capsid protein L1 epitope of HPV types 16 and 18	Insect cells
Sci-B-Vac™	Hepatitis B Virus	Hepatitis B	Approved	VBI Vaccines	The three epitopes of hepatitis B surface antigen: S, Pre-S1, and Pre-S2	Eukaryotic cells (Chinese hamster ovary (CHO) cells)
Mosquirix™	*Plasmodium falciparum*	Malaria	Approved	Glaxo-SmithKline Inc	Plasmodium falciparum circumsporozoite protein fused to the Hepatitis B surface antigen, combined with Hepatitis B surface antigen (S)	Yeast
Still no trade name	Influenza Virus	Seasonal Flu	Clinical trial Phase 3 (Clinical trial No.: NCT03301051)	Medicago	A mix of recombinant H1, H3, and two B hemagglutinin proteins	Plant (*Nicotiana benthamiana*)
Still no trade name	Influenza Virus	Pandemic Flu	Clinical trial Phase 2 [24]	Medicago	Hemagglutinin protein	Plant
Still no trade name	SARS-CoV-2 Virus	COVID-19	Clinical trial Phase 1 (Clinical trial No.: NCT04450004) [25]	Medicago	SARS-CoV-2 spike protein	Plant (*Nicotiana benthamiana*)
NanoFlu™	Influenza Virus	Seasonal Influenza	Clinical trial Phase 3 (Clinical trial No.: NCT04120194) [26]	Novavax	Recombinant hemagglutinin (HA) protein	Insect cells
ResVax™	Respiratory Syncytial Virus (RSV)	RSV	Clinical trial Phase 3 [27]	Novavax	RSV fusion (F) protein	Insect cells
NVX-CoV2373	SARS-CoV-2 Virus	COVID-19	Clinical trial Phase 1 [28]	Novavax	Trimeric full-length SARS-CoV-2 spike glycoproteins	Insect cells
Ebola GP Vaccine	Ebola Virus	Ebola	Clinical trial Phase 1 [29]	Novavax	Ebola glycoprotein	Insect cells
Hecolin®	Hepatitis E Virus	Hepatitis E	Approved [30]	Xiamen Innovax Biotec	A segment (amino acids 368–606) of the HEV open reading frame 2 (ORF2) capsid protein from HEV genotype 1	*Escherichia coli*
Still no trade name	HIV (human immunodeficiency virus)	Acquired immunodeficiency syndrome (AIDS)	Clinical trial Phase 1 (Clinical trial No.: NCT00001053) [31]	Univ. of Rochester AVEG, Rochester, New York, United States	HIV p17/p24:Ty-VLP	Yeast
Still no trade name	Norwalk Virus	Acute Gastroenteritis	Clinical trial Phase 1 (Clinical trial No.: NCT00973284) [32]	Takeda	Derived from norovirus GI.1 genotype	Insect cells

## Structural classification of VLPs

**Fig. 1:**
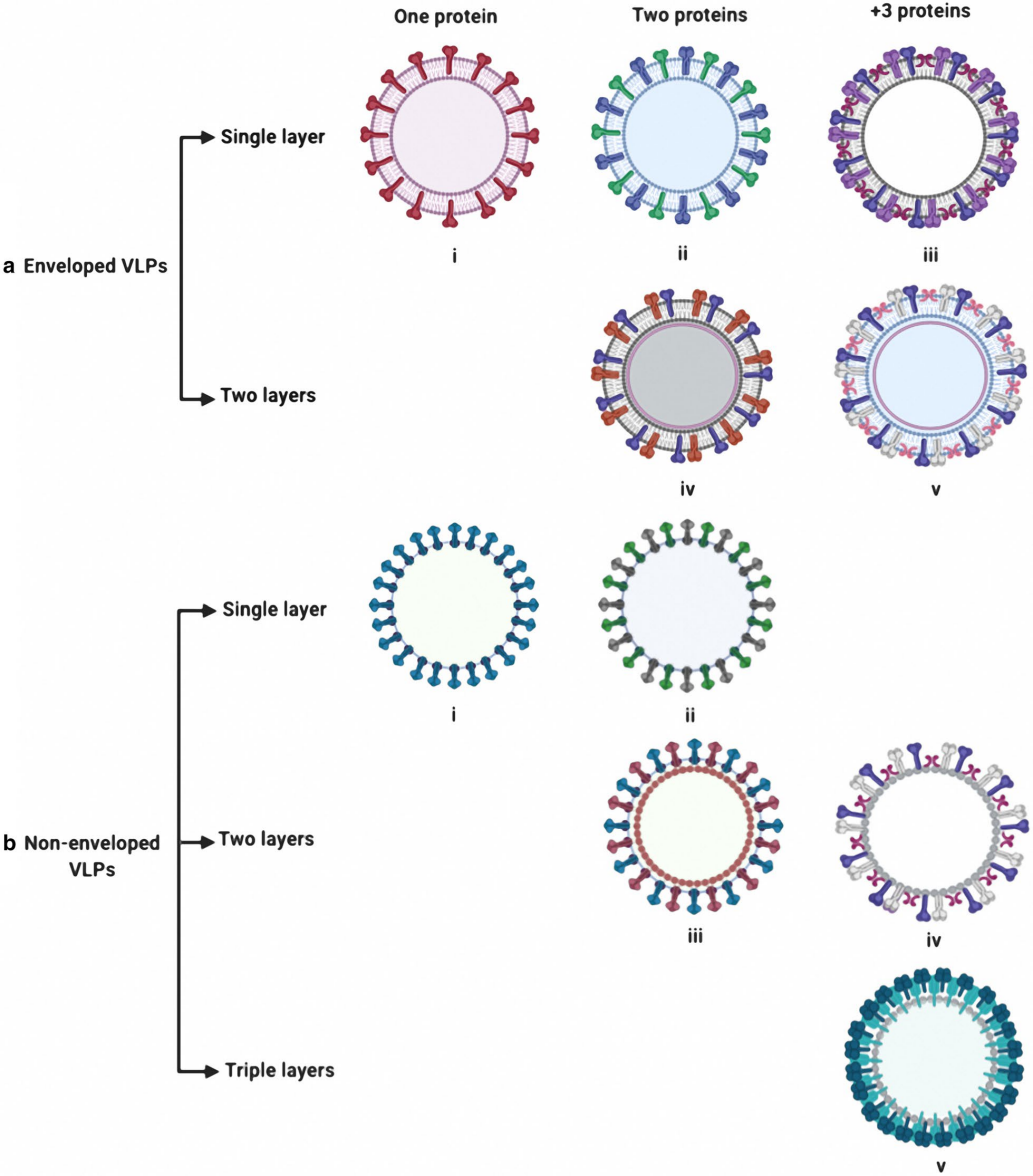
Classification of various VLPs structure. **a** For enveloped VLPs, expression of one (i) or two glycoproteins will form a single layer, as demonstrated by the expression of influenza virus hemagglutinin alone or co-expression of both hemagglutinin and neuraminidase, respectively. Expression of more than three glycoproteins (iii) will also form a single layer VLP. Double layered enveloped VLPs can be formed by the multiple glycoproteins on their surface that can have two (iv) or more than three glycoproteins (v). **b** For non-enveloped VLPs, single layered non-enveloped VLPs can be assembled from a single protein (i) or two proteins(ii). Double layered non-enveloped VLPs can be assembled from two proteins (iii) or more than three proteins (iv). Triple layered VLPs (v) have been assembled from more than three proteins

VLPs are formed by spontaneous interaction between one or more viral structural capsid proteins to form the final structure. VLPs are structurally and visually similar to live viruses but lack either a complete virus genome or lack the entire virus genome. The variety of structures adopted by different VLPs makes them structurally and functionally attractive. Spontaneous polymerization of different viral capsid proteins can produce VLPs with geometrical symmetry, usually in the form of icosahedral, spherical, or rod-like structures, depending on which virus from which they were derived. VLPs can generally be divided into different groups based on their structural complexity. Capsid proteins can be arranged in one, two or three layers. Other single layer VLPs can contain more than one structural protein. While single-protein VLPs have a relatively simple structure, multi-protein VLPs contain unique structural components such as presence of several distinct capsid layers (Fig. 1a). Other VLPs, such as those derived from HIV-1 and influenza virus, have a layer of lipid that contains viral surface antigens surrounding the capsid structure, reflecting the lipid envelope found in the natural infectious virus particle. The presence or absence of the envelope provides an additional structural classification for VLPs (Fig. 1b). Frequently enveloped VLPs contain a matrix protein located immediately inside the host-derived lipid membrane in which the viral glycoproteins embedded. The requirement for generation of a lipid envelope and the targeting of virus proteins to the lipid bilayer for some VLPs places requirements on the choice of production system that can be used for their generation [33].

### Non-enveloped VLPs

Non-enveloped VLPs are further classified as single or multi-capsid protein VLPs and also as single-layer, double-layer, and triple layer VLPs. The simplest available non-enveloped model of VLP consists of single capsid VLP structure like human papillomavirus (HPV) VLP vaccines. These simple VLPs are composed of a single capsid protein that can be expressed in both eukaryotic and prokaryotic systems. In some cases of the simple VLPs, capsid proteins can also be formed in a cell-free system. In these cases to form soluble homogeneous VLP capsomer the proteins can be expressed first in a cell-based expression system and then assembled in a cell-free environment for proper folding [34–37]. In contrast, production of the multi-capsid non-enveloped VLPs are more complex and challenging. These complex VLPs are usually made in eukaryotic expression systems such as yeast [38, 39], insect cells [40] and plants [41]. Different capsid proteins can be expressed and assembled in one cell. Examples of such multi-capsid proteins VLPs that have been successfully assembled into multiple layers and produced in a heterogeneous hosts include VLPs of bluetongue virus [42], Enterovirus 71 [43, 44], infectious bursal disease virus [45], poliovirus [46] and rotavirus [39, 47]. Another examples of the multicapsid non-enveloped VLPs including papillomavirus L1 and L2 protein that can be assembled into VLPs from two proteins [48].

### Enveloped VLPs (eVLPs)

Enveloped VLPs are also sub-divided into single-layer, double-layer and multi-layer internal structures that lie below the lipid envelope. Enveloped VLPs obtain their lipid membrane from the cell in which they are expressed during the assembly and budding of VLPs from the cell. One or more glycoprotein anchors can be inserted into the lipid membrane, and these glycoproteins are commonly the main target antigens detected by the immune system for the production of neutralizing antibodies. The precise nature, origin and composition of the envelope are different for various enveloped VLPs and the precise detail of their nature depends on the virus from which the VLP is derived which in turn determines the process of assembling and budding of the VLP from the host cell line that is used for their production.

### Challenges and solutions for developing eVLP-based vaccines

Formation of fully infectious virus particles requires the presence of all of the necessary components including the viral genome, internal structural proteins, and, if present, the glycoproteins, but the minimum requirements for eVLP formation are not well understood [49]. Typically, self-assembly of enveloped VLPs involves two steps including the formation of an internal protein (nucleocapsid, and/or matrix) and then acquisition of the membrane. The assembly and ultimate release from the cell of eVLPs may be dependent on internal viral structural proteins, envelope glycoproteins, or both [50]. In retroviruses, for example, expression of the gag nucleoprotein alone can lead to eVLP formation, but in coronaviruses and flaviviruses expression of glycoproteins leads to the release of eVLPs, that are morphologically similar to whole [51]. Proper folding and glycosylation of these viral surface antigens, plays an important role in the efficacy of eVLP-based vaccines because it is critical for stability, immune recognition, and pathogenicity of these antigens [52]. For instance, a mutant Zika virus which lacks N glycosylation of E protein is attenuated in mammalian and mosquito hosts [53]. Glycosylation of E protein is also a major determinant of the viral pathogenesis of the dengue viruses (DENV) [54]. Technical challenges remain in the generation of eVLP-based vaccines in terms of design, treatment, and storage. Stability of the eVLP-based vaccine is one of the most important issues [55]. In general, eVLPs that have a host-derived envelope are more sensitive to the external environment than non-enveloped VLP that are comprised of only a protein capsid. Changes in conditions such as temperature, shear force, and process that are used in purification can destroy the integrity and stability of particles with an associated reduction in immunogenicity of eVLPs. In some cases, VLPs have been modified to improve their temperature resistance by the introduction of stabilizing mutations [56]. A problem that can arise lies in the observation that the expression of viral proteins varies significantly in different systems. In general, the expression of glycoproteins is difficult and if this presents a limitation on the production of eVLPs into the cell medium cell lysis or other extraction steps may be required which imposes more costs and steps for further refinement. A common approach to improve expression level of the secretory glycoproteins is to remove or replace the transmembrane region that anchors the protein in the membrane. Replacement of the stem construct of the DENV2 E protein ER retention signal with the corresponding region of Japanese encephalitis virus (JEV) provided extracellular secretion of eVLPs [57]. This is not always successful as the membrane integration-dependent oligomerization of glycoproteins is sometimes required for full functionality and that can be adversely affected by the introduction of chimeric sequences. Another way to potentially improve eVLP secretion is to introduce a suitable heterologous signal peptide. The signal peptide is one of the most important factors affecting downstream proteins transport and topology, though there is a risk that introduction of an exogenous signal peptide sequence may alter protein properties [58]. Finally, during a large-scale production and purification (LSP) the presence of impurities associated with the eVLPs represents a daunting challenge. Contaminants are usually due to features of the host cell and take the form of cell debris, host cell proteins, DNA, and lipids. In the baculovirus expression system (BVES) for example, biophysical properties, such as size, electrostatic charges, and architecture, complicate LSP for eVLPs which can generate undesirable side effects in the vaccinees [59]. The purity of the final product is a critical issue and in some situations further treatments may be required to reduce the levels of unwanted contaminants, but there is a risk that these may also affect the antigenicity of eVLPs itself.

## Production, purification and formulation of VLP vaccine

**Fig. 2:**
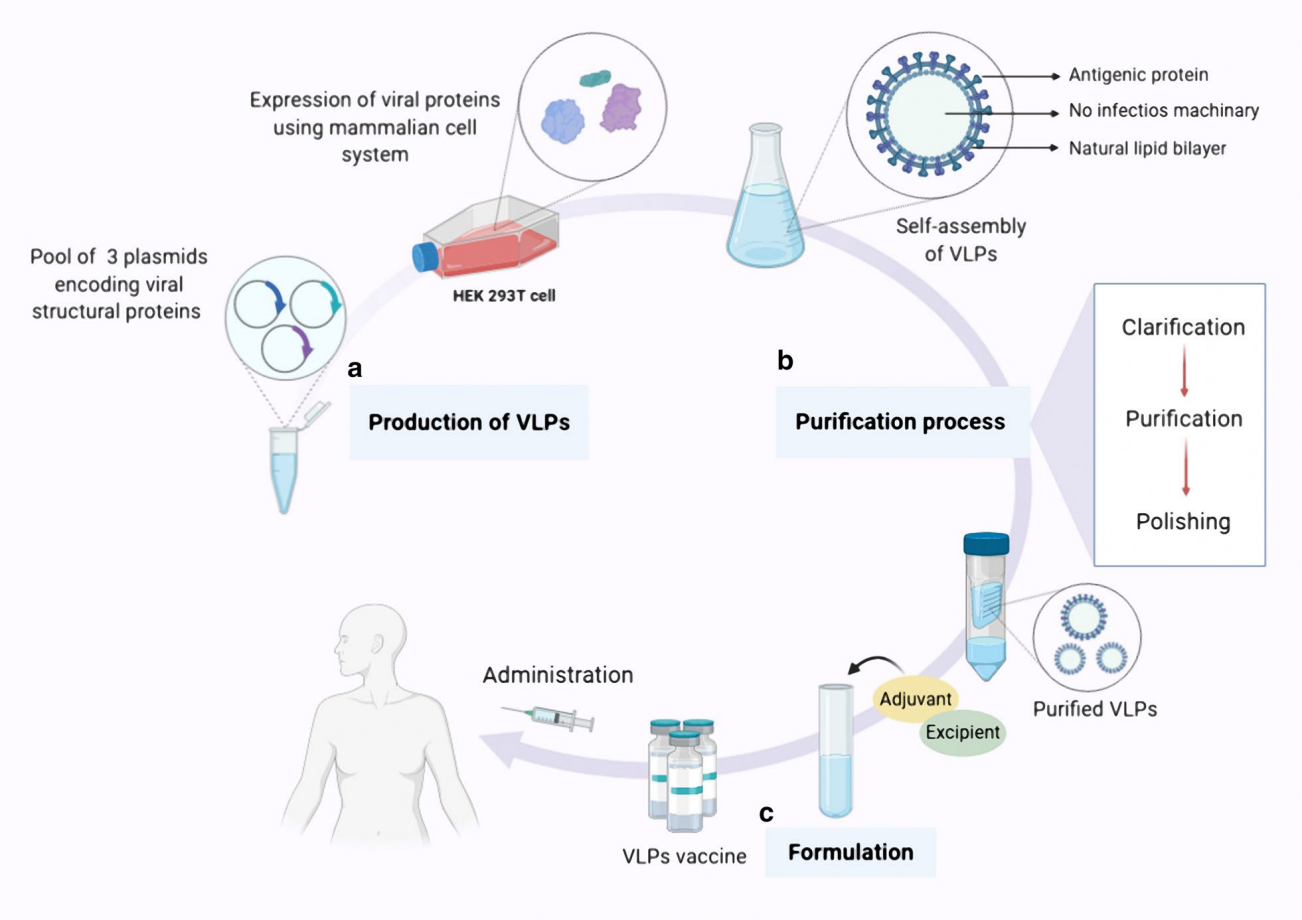
Overview of VLP-based vaccine expression, purification and formulation. In general, the process of manufacturing VLP-based vaccine consists of three stages. **a** Production stage; which includes cloning of the viral structural genes of interest and expression of viral proteins with self-assembling ability in a suitable expression platform (The HEK293T cell line, a mammalian expression system, is shown here) At the end of this stage, the VLPs are collected in the form of particles that do not have infectious properties. **b** Purification stage which briefly consist of downstream processing such as clarification, purification and polishing to finally obtaining purified intact VLPs with no residual host debris. **c** Formulation stage; in which adjuvant and additional ingredients are added to the vaccine formulation to finally achieve a safe, efficient and effective product for vaccination

The generic manufacturing process for a VLP-based vaccine generally consists of three main sections: (A) upstream processing (production), (B) downstream processing (purification), and (C) formulation. The first step in VLP production is to clone the viral structural genes of interest. Next, viral structural proteins with self-assembling ability are expressed in prokaryotic (bacteria, yeast) or eukaryotic (baculovirus/insect cell, mammalian cell and plant) expression systems. After harvesting and lysing the cells, to ensure removal of contaminating cell debris and aggregates a clarification step is performed [55, 60]. To obtain intact and more purified VLPs, further purification steps such as ion-exchange chromatography and ultracentrifugation are needed [60]. A final purification step, polishing, is used to remove the residual host cell proteins and nucleic acids [55, 61]. In the last step of manufacturing process of VLPs vaccine development, sterile filtration and formulation is done to finally achieve a safe, efficient and effective product (Fig. 2) [55].

### Expression platforms for producing VLPs

Various expression platforms including prokaryotic, and eukaryotic systems can be used for producing VLP vaccines [62, 63]. Eukaryotic systems that have been used include the *baculovirus*/insect cell (B/IC) system [64], mammalian cell culture [65] and plants [41]. In addition cell-free expression systems have also been used successfully. Choosing an appropriate expression system to produce VLPs is a crucial factor to ensure proper protein folding and post-translational modifications (PTM) [66]. The quaternary structure of viral capsid proteins can differ in different expression systems due to protein PTMs such as glycosylation and phosphorylation and this may affect the immunogenicity of the vaccine as PTMs are often necessary to stimulate an appropriate immune response [67]. Each expression system has benefits and drawbacks which are briefly highlighted below.

#### Bacteria

Bacteria are one of the most widely used expression systems for the production of recombinant proteins and are also used to produce many VLPs. However, due to various factors such as lack of PTM system, incomplete disulfide bond formation and protein solubility problems, they are not suitable platforms for producing enveloped VLPs [68]. However, bacteria are a suitable expression system for generating of non-enveloped VLPs with one or two viral structural proteins [66]. In vaccine development, the high cost of the product would limit vaccine utilization, especially in developing countries. For these reasons a prokaryotic-based expression system is often seen as the best for development of VLPs vaccines due to the ability to produce safe and cost-effective vaccines for global use [69].

*Escherichia coli* is the most common bacterial host cell for VLP production [68]. An *E. coli* expression system has many advantages including low production cost, rapid cell growth, high protein expression level, and simplicity of scaling-up. The *E. coli* expression system is commonly suggested for producing of small proteins with limited PTM [69]. Various VLP vaccines generated using *E. coli* expression systems have entered clinical trials for use against infectious and non-infectious diseases. Hecolin, a Hepatitis E vaccine manufactured by Xiamen in the form of a p239 VLP-based vaccine, was the first Hepatitis E virus (HEV) approved vaccine using an *E. coli* expression system [30]. An *E. coli* expression platform to produce a bivalent vaccine against HPV 16/18 L1 VLPs has also been shown to be safe and immunogenic [69, 70]. Malaria vaccine (MalariVax), a chimeric VLP-based vaccine which is comprised of two fused proteins, the core proteins of HBV and epitopes of circumsporozoite proteins of *Plasmodium falciparum*, has been successfully produced in *E. coli* [71–73]. In addition, the M2e-HBc VLP-based candidate vaccine which used an *E. coli* expression system for self-assembly has shown complete protection in mice against influenza [74]. Many other VLPs based vaccine candidates using *E. coli* expression system against various infectious such as West Nile virus (WNV), foot-and-mouth disease (FMS) virus, and HCV have also entered preclinical trials [69]. In addition to *E. coli* the successful formation of VLP has been observed in some other bacterial species. Self-assembly of HPV-16 L1 protein VLPs was successfully carried out in *Lactobacillus casei* using a lactose-inducible promoter system for L1 protein expression [75]. The cowpea chlorotic mottle virus (CCMV) coat proteins, have also been successfully expressed in *Pseudomonas fluorescens* [76].

Furthermore, several chimeric VLPs vaccines against non-infectious diseases including hypertension, allergies, diabetes, cancer, and Alzheimer's have been sufficiently developed by antigen conjugation with bacteriophage Qβ RNA in *E. coli* expression platform [69].

#### Yeast

Yeast cells are frequently used for recombinant proteins expression and has also been used for VLPs production [67]. Yeast expression platforms, especially *Saccharomyces cerevisiae* and *Pichia pastoris*, are most favored due to advantages such as rapid cell growth, high yield of expression proteins, scalability, cost-effective production, and providing a degree of PTM processes [67, 77]. Two FDA-approved VLPs–based vaccines, Engerix-B (HBV vaccine) and Gardasil (HPV vaccine) have been generated in yeast expression systems [78, 79]. Recently, the production of Chikungunya VLPs (CHIK-VLPs) using *Pichia pastoris* has been reported [80]. Despite these achievements, the lack of complex PTM pathways is a major drawback of yeast expression systems, which limits their use for VLP production. Additional issues are the potential of high mannose glycosylation, plasmid loss and lower yields of protein compared to bacterial expression system can be other issues, which should be considered [77]. The yeast-based systems are therefore generally used for generating non-enveloped VLPs. However, yeast systems have been used successfully to HIV 1 Gag protein VLPs and DENV-2 VLPs [81].

#### Baculovirus/Insect cells (B/IC)

The B/IC expression system is the most commonly used expression system for production of both enveloped- and non-enveloped-VLPs [77]. Due to the convenience and speed of baculovirus-based VLP expression, this system is suitable for manufacturing vaccines against viruses that are rapidly changing their surface antigens between each outbreak such as influenza virus [66]. Insect cell expression systems have several advantages for VLP production such as high yield of expressed proteins comparable to those obtained from bacteria or yeast, the presence of complex PTM pathways and formation of multi-protein VLPs [67]. The conventional insect cell lines used for producing of recombinant proteins are derived from *Spodoptera frugiperda* (Sf9/Sf21) and *Trichoplusia ni* (Tn5) [77]. Cervarix, the FDA-approved HPV vaccine, consisting of HPV16 and HPV18 L1-protein-based VLPs has been produced using this expression system. The *Baculovirus*/insect cell platform has also been used for obtaining prophylactic vaccine candidates against several infectious diseases such as HIV 1, influenza virus A, Chikungunya virus, severe acute respiratory syndrome (SARS), Ebola virus, dengue fever virus, Rift Valley fever virus (RVFV), Norwalk virus and HCV [77, 82]. The main potential drawback of the baculovirus/insect cell platform is the simpler N-glycosylation pattern for the expressed glycoproteins when compared to mammalian cells, which can be a disadvantage for some VLP applications [83]. However, an effective strategy based on enhancing the insect cell N-glycosylation machinery by improving glycosylation pathways of certain insect cells, such as Ea4 has been shown to simplify production of the therapeutic human glycoproteins [84]. Therefore, if the insect cell glycosylation pattern improves, the B/IC system is probably the strongest candidate expression system for VLP-based vaccine manufacturing [55].

#### Plants cells

Plants expression systems have several advantages over conventional approaches, including high expression levels of up to 80% total soluble protein, low refining costs, and high-performance expression processing. Using MagnICON and CPMV-HT technology, plant-based vaccine has become a versatile and promising platform bringing the cost of protein production to less than $50 per gram for the production of several proteins and antibodies used in the human and veterinary pharmaceutical industry which [85–87]. The long history of study of tobacco mosaic virus (TMV) [88], with genome analysis [89], determination of the three-dimensional (3D) structure of the virus particles and identification of surface region exposed by structural studies, were milestones in the development of recombinant plant-based vaccines [90]. Several experimental vaccines based on noninfectious plant VLPs have been successfully constructed. More than 55 different plant viruses have been used to create a platform for antigen expression on their surface, including TMV, cucumber mosaic virus (CMV), alfalfa mosaic virus (AIMV), cowpea mosaic virus (CPMV), papaya mosaic virus (PapMV), and the potato X virus (PVX). Of these TMV, PVX, CPMV and CCMV are more stable at high temperatures and pH and are expressed in large quantities in native plants host [91]**.**

Detection of 34 different plant viruses from the human gastrointestinal tract and monitoring of some plant viruses in human feces showed that plant viruses such as CPMV and pepper mild mottle virus (PMMoV) are structurally very stable in intestinal conditions [92]. Antigens displayed on the plant VLP surface undergo interaction with antigen-presenting complexes (APCs), followed by activation and increase in the number of tumor infiltrating neutrophils and also contributes to activation of dendritic cells (DCs). The empty CPMV capsid used as a VLP, or complete CMPV virions containing genomic RNA, have been shown to be able to induce immunomodulatory activities including demonstration of in situ antitumor immune activity resulting in tumor regression [93]. The ability of TMV particles to bind to up to 140 plasma proteins and immunoglobulins has made them the most widely used model in biological studies and plant-based vaccine research. Over recent years more than 100 experimental vaccines have been developed for human and veterinary diseases using plant viruses against a wide range of diseases, including cancer, infectious diseases and autoimmune diseases [94–96]. Two replicon systems in particular have been shown to induce strong expression of VLP-based vaccines in plants including deconstructed viral vectors composed of TMV RNA replicon system (MagnICON) and the Geminiviral BeYDV DNA replicon system [85, 97].

Plant systems have also been used to generate VLPs based on animal and human viruses. Induction of Norwalk virus (NV)-specific intestinal mucosal antibody requires administration of an oral vaccine. Non-enveloped Norwalk virus capsid (NVCP) VLP has been successfully expressed in tomatoes, potatoes, tobacco and lettuce with a structure similar to native NV particles replicated in the human gastrointestinal tract. However, these systems display slow and very low expression and accumulation of NVCP VLP which has been a major hurdle to their use. To overcome these limitations, new developments in an expression platform based on the BeYDV and MagnICON replication systems have led to an increase of more than 80-fold in the accumulation of NVCP VLPs in transgenic tobacco and tomato [98].

More recently the use of plant-based systems to express heteromultimeric protein complexes which resemble influenza VLPs has been explored with positive indications that good yields can be achieved from plants in a matter of days.The cowpea mosaic virus-Hyper Translatable (CPMV-HT) platform, which is based on the rapid transient expression of recombinant proteins in plants through Agrobacterium-mediated infiltration, has been used to produce 10 M dose of VLP H1N1 (swine flu) vaccine in just 30 days compared to the 9–12 months required for more common approaches in current use [24, 86]. In addition, complete assembly in a plant VLP system of four African horse sickness (AHS) capsid proteins which mimic the structure of the native virus, resulted in spontaneous assembly of a VLP using the CPMV-HT expression system in *Nicotina bentamiana* [99]. These VLPs were able to induce a strong immune response against AHS.

There are several licensed Yeast and Chinese hamster ovary (CHO) cells-derived VLP-based vaccine against hepatitis B virus surface antigens (HBsAg) [22]. In addition, small HBs surface antigen (S-HBsAg) molecules were assembled into VLPs, successfully expressed in plants (lettuce, potato and lupine) and administered as an edible vaccine. These VLPs were structurally similar to the licensed yeast-derived vaccine and were strongly immunogenic [91, 100, 101]. A malaria transmission-blocking vaccine consisting of the Pfs25 surface protein from *Plasmodium falciparum* parasite conjugated to the AIMV has been generated. This plant-based VLP, Pfs25 VLP-FhCMB, was produced by transient expression in *N. bentamiana* and its safety and immunogenicity in phase I clinical study was successfully evaluated [102]. Medicago has developed a platform for production of plant-based vaccines against several viral pathogens including coronavirus, rotavirus and norovirus. The Medicago’ norovirus-like particle vaccine candidate which mimics the native norovirus is currently being evaluated in pre-clinical studies. A plant-derived quadrivalent VLP candidate as an influenza vaccine was able to induce both strong antibody and cellular responses in the first phase III efficacy study and is currently being finalized by Health Canada. A similar approach has generated a plant-based Coronavirus VLP-based vaccine candidate which is undergoing Phase I trials. It is estimated that this approach could produce approximately 100 million doses of plant based COVID-19 vaccine by the end of 2021 [103].

#### Mammalian and avian cells

Animal cell expression systems remain valuable and attractive platforms which can be used for producing multiple structural proteins of non-enveloped and enveloped VLPs [66, 77]. Animal cell expression platforms are the most efficient systems for recombinant protein production due to their ability to make complex and precise PTMs that are essential for proper protein folding [68]. Several animal cell lines, including CHO, baby hamster kidney-21 (BHK-21), human embryonic kidney 293 (HEK293), CAP‐T cell line derived from human amniocytes, Vero 9, and east lansing line-0 (ELL-0) are extensively utilized for production of recombinant VLPs [66]. CHO, the most frequently used cell line, has the advantage over other cell lines that it is not derived from human cells and therefore it has a lower risk of contamination with human viruses [66].

Intracellular assembly of HBsAg VLPs using the CHO cell line is an example of a very successful production system and both dengue virus VLP and Hantavirus VLP have also been generated in CHO cells [104, 105]. The HEK293 cell line has been used to produce VLPs for use against HIV, influenza, and rabies viruses and the CAP-T cell line has also been shown to be a highly efficient expression system for HIV VLPs production [106]. In a recent study on developing a candidate VLPs vaccine for protection against the newly emerged disease COVID-19, a stable SARS-CoV-2 VLP has been produced, using the Vero E6 cell line [107]. In addition to mammalian cell lines, avian cell lines have also been used to produce VLPs. Newcastle disease virus (NDV) and VLPs consisting of F and G proteins of respiratory syncytial virus (RSV), as a VLPs vaccine candidate against the human RSV has been generated from avian ELL-0 fibroblast cell line [108]. However, low protein yield, high production cost, long expression time and the possibility for cell lines to carry infection with mammalian pathogens are considered to be major potential disadvantages of mammalian cell expression systems for generating material for clinical use [77].

#### Cell-free system

Cell-free protein synthesis (CFPS) systems provide an additional option for the in vitro expression express the recombinant proteins for VLPs production [109]. These systems are typically composed of bacterial or yeast cells for the synthesis of virus capsid proteins [67]. CFPS systems have many advantages compared to cell-based protein expression platforms such as time-saving, high yield of proteins, limited cellular contaminants and the option of generating VLPs containing unnatural amino acids (UAAs) or toxic protein intermediates [67, 109]. However, these expression systems have significant limitations for commercial application including very high production cost and limited scalability [67]. Despite the drawbacks, such systems have been used and two examples of commercially utilized vaccines are Inflexal, an influenza VLP vaccine, and the Epaxal hepatitis A VLP vaccine. Norovirus and Hepatitis B VLPs have also been efficiently expressed by using in vitro expression systems [109].

### Purification of VLP-based vaccines

Downstream processing for VLP purification is a crucial step to ensure suitable efficacy and safety for clinical use [55]. After cell harvesting, the nature of the first stage in the purification process depends on the ability of the VLPs to be released into the extracellular medium. While in some reported cases such as influenza VLPs produced in insect cell culture the particles are released into the medium without the need for special measures, if the VLP is not released effectively, cell lysis or an alternative extraction method may be required to disrupt the cells [110]. The generally adopted approach is to design a cloned gene to express a protein containing an effective signal peptide that will be recognized by the secretory pathway to facilitate release [55, 60].

To reduce the steps and costs of the purification process, a clarification step is performed to remove whole cell debris and aggregates from primary VLP preparations. Capturing and concentration of VLPs is a critical step that significantly reduces the bulk volume and increases the ratio of VLP concentration to other cellular impurities. A variety of methods is used for clarification, including cell sedimentation, depth filtration and microfiltration tangential flow filtration (TFF). For separation of the VLPs from host cell contamination like cell debris, digested DNA or components of media, ultrafiltration/diafiltration (UF/DF) and TFF with membranes or hollow fibers methods are commonly used. Affinity chromatography, ion-exchange chromatography, and hydrophobic interaction chromatography may also be used for capturing VLPs in bind-and-elute approach [101, 102, 111]. Other intermediate purification steps such as IEC, HIC, and Super centrifuge are often required to reduce DNA and endotoxin levels.

Disassembly and reassembly is an optional step which is performed to increase stability, homogeneity and immunogenicity of VLPs product. Titration or cross-flow filtration is a chosen option for this step [55]. In the final purification step, referred to as the polishing step, all residual contaminants arising from the processing of VLP must be removed. This is commonly done by IEC, size exclusion chromatography (SEC) and, UF/DF (usually cross-flow method) [55, 111]. Prior to final formulation the preparations are sterilized by filtration through 0.22 μm sterile-grade filters [111].

### Formulation of VLP-based vaccines

Vaccine formulation focuses on improving the stability, efficacy, and safety of the vaccine during storage and shipping until the point of administration. For optimizing the efficacy of VLPs adjuvants and authorized excipients are added in most vaccine formulations [20, 112]. In order to protect VLPs from physical and chemical instability and enzymatic degradation, excipients such as buffers, preservatives and other stabilizing chemical compounds are added for the final product formulation. Additives such as polysorbate 80, l-histidine, sodium borate/phosphate and 2-phenoxyethanol are often used in the formulation of VLP-based vaccines as a surfactant stabilizer, buffering agent and preservative, respectively [112]. The addition of carbohydrate preservatives, such as glycerol, sucrose, and trehalose to vaccine formulations has been shown to increase the stability in liquid suspension of Norwalk and rotavirus VLPs. Study of the effects of addition of a polyanion to CHIKV VLPs that are unstable at neutral pH showed that all polyanions investigated were able to stabilize CHIKV VLPs against aggregation.

Many VLPs have molecular and structural properties that can spontaneously stimulate the immune system, thus eliminating the need to use adjuvants. Nevertheless, using adjuvants in VLPs vaccine formulations may increase the immunogenicity of the vaccine and stimulate a specific type of immune response [112]. Several classes of adjuvant have been tested for VLP vaccines such as; aluminum salt-based (Alum) adjuvant, liposome/virosomes, pattern recognition receptors (PRRs) agonist adjuvant, chitosan, emulation adjuvant, interleukin 12 (IL12) and, bacterial toxin [19]. Aluminum salts are the most well-known adjuvant used in vaccine formulations and these have also been used in the formulation of all approved VLPs vaccines [112]. Insoluble aluminum salts such as aluminum phosphate, aluminum hydroxyl-phosphate, and aluminum hydroxide are used for preparation of Alum-based vaccine. Engerix-B (HBV vaccine), Gardasil (HPV vaccine), Cervarix (HPV vaccine), and Hecolin (HEV vaccine) are commercialized VLP-based vaccines which are formulated with aluminum salts adjuvant [111]. Inflexal (Influenza vaccine) and Epaxal (HEV vaccine), are two commercialized virosomal-based adjuvanted VLPs vaccine, which have shown significant efficacy and safety in various studies [109].

PRR agonist adjuvants such as AS04, AS01/AS02, Poly I:C/Poly ICLC (double-stranded RNA analogs), CpG oligonucleotide (ODN), flagellin, Toll like receptors 7 (TLR7), and TLR7/8 agonists, which are compounds derived from pathogen-associated molecular patterns, are the amongst the most commonly used adjuvants for VLP formulation [19, 112]. The AS04 adjuvant, a licensed TLR4 agonist adjuvant consisting of MPLA (3-O-desacyl-4′-monophosphoryl lipid A) in combination with aluminum phosphate or hydroxide salts are used in the Cervarix VLP-based HPV vaccine to enhance the immune response in vaccine. In a recent study using filovirus VLPs as a model vaccine to evaluate several candidate vaccine adjuvants including poly-ICLC, MPLA, TLR4, CpG ODN2395 and alhydrogel, the results showed that the use of poly-ICLC as an adjuvant for enhanced long-term protection against the Ebola virus is effective [113]. TLR7 and TLR7/8 agonist adjuvants are effective VLPs adjuvants that have been shown to be able to directly activate APCs and have the ability to induce humoral and cellular immune responses [19]. In a preclinical study performed on the HPV vaccine based on L1 protein and VLPs it was shown that a TLR7 agonist adjuvant in the formulation led to good induction of appropriate neutralizing antibodies against HPV16 [74]. In addition to the use of TLR agonist adjuvants in VLP-based vaccines to prevent infectious diseases, some TLR adjuvants have been used in the context of anti-cancer vaccination. Clinical trials in several metastatic malignancies revealed that TLR3 can stimulate immune responses to levels that provide clinical benefit and prolonged survival of patients. Imiquimod, a type of TLR7 agonist adjuvant, is currently approved to be formulated with both a melanoma VLP vaccine, and a noninvasive bladder cancer VLP vaccine [114].

Chitosan is a mucosal adjuvant that can effectively deliver the vaccine to local phagocyte cells and thus has the ability to stimulate strong induction of both systemic and mucosal immune responses [19]. Intranasal administration of a norovirus VLP-based vaccine formulated with chitosan has been shown sufficiently provide effective immunization against Norwalk viral gastroenteritis and infection [32]. Formulation of influenza VLPs vaccines with MF59 (an emulsion adjuvant) has shown a high level of safety and efficacy. In a similar approach, formulation of a RSV VLP-based vaccine with AddaVax (InvivoGen), which is analogous to MF59, has been demonstrated to elicit elevated levels of neutralizing antibody and enhanced immune response [115].

One of the important cytokines involved in immounoregulation which is produced primarily by antigen-presenting cells is IL-12 [19]. In an animal study it has been shown that a VLP vaccine against influenza (H3N2) formulated with IL12 as an adjuvant led to enhanced antibody responses and that protected 90% of the mice against a lethal influenza virus infection [116]. Cholera toxin (CT) and heat-labile toxin (LT), have been shown to be adjuvants that have ability to enhance the immune responses [19]. A study in which an influenza VLP-based vaccine formulated with CT showed that the vaccine led to a reduction in viral load in the lungs of infected mice, and additional study showed that using either B subunits of CT or LT of *E. coli* as an adjuvant with a rotavirus VLP vaccine efficiently enhanced specific immune responses [117, 118].

### Characterization of VLPs

**Fig. 3:**
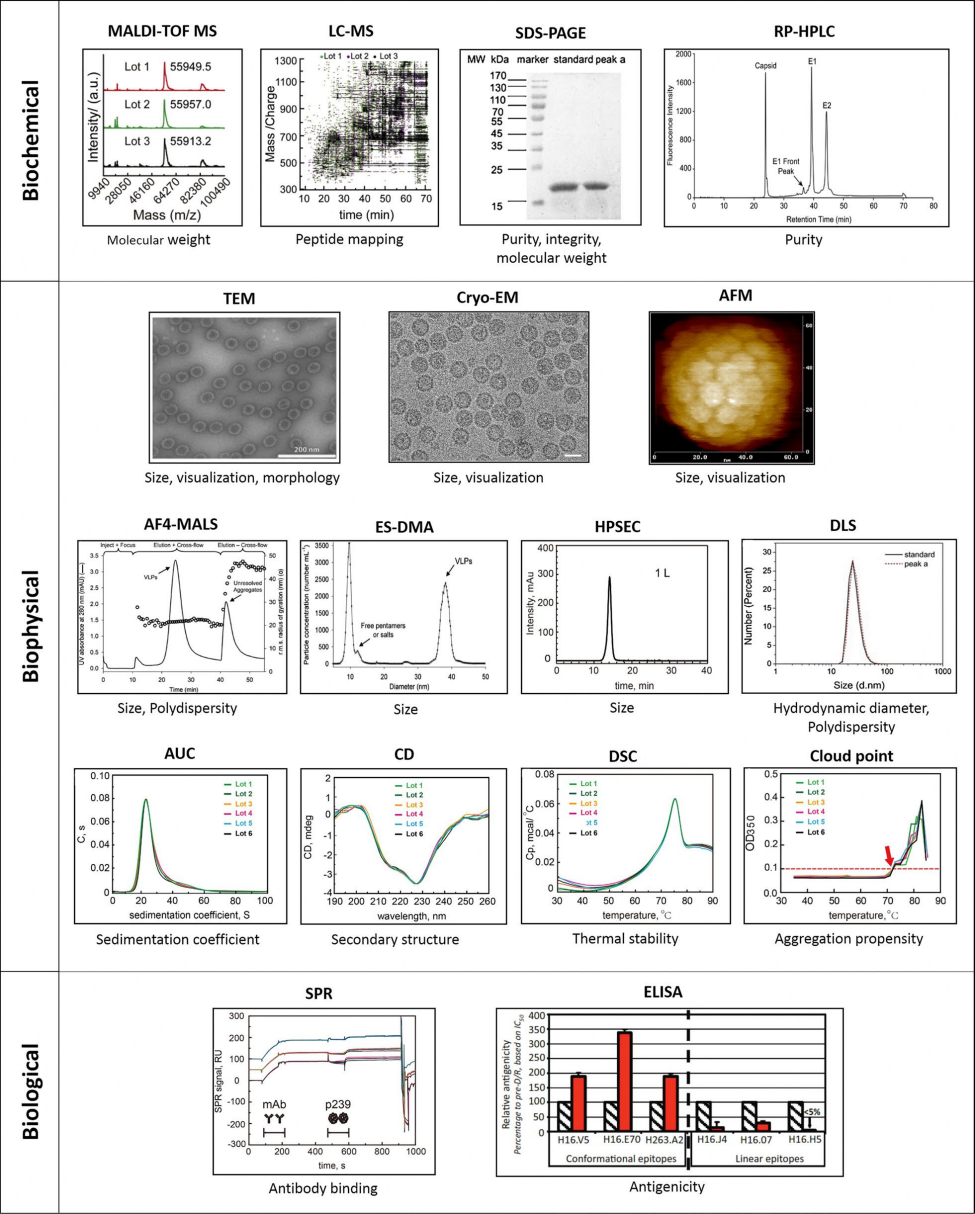
Analytical techniques for characterization of VLPs. A series of analytical tools has been used for biochemical, biophysical and biological characterization of VLPs. Biochemical method: Matrix assisted laser desorption ionization time of flight-mass spectrometry (MALDI-TOF MS), liquid chromatography–mass spectrometry (LC–MS), sodium dodecyl sulphate polyacrylamide gel electrophoresis (SDS-PAGE) and reverse phase-high performance liquid chromatography (RP-HPLC). Biophysical method: Transmission electron microscopy (TEM), cryo-electron microscopy (Cryo-EM), atomic force microscopy (AFM), asymmetric flow field-flow fractionation coupled with multiple-angle light scattering (AF4-MALS), electrospray differential mobility analysis (ES-DMA) and high performance size exclusion chromatography (HPSEC), dynamic light scattering (DLS), analytical ultracentrifugation (AUC), Circular dichroism (CD), differential scanning calorimetry (DSC), Cloud point. Biological characterization: Surface plasmon resonance (SRP), enzyme-linked immunosorbent assay (ELISA). MALDI-TOF MS and LC–MS images are reprinted with permission from Elsevier, Reference [119]. SDS- PAGE and DLS images are reprinted with permission from Elsevier, Reference [121]. RP-HPLC image is reprinted with permission from Elsevier, Reference [122]. TEM, A4-MALS and ES-DMA images are reprinted with permission from John Wiley and Sons, Reference [127]. Cryo-EM image is reprinted with permission from Elsevier, Reference [123]. AFM is reprinted with permission from Elsevier, Reference [125]. HPSEC, AUC, CD, DSC, Cloud point and SPR images are reprinted with permission from Elsevier, Reference [128]

Biochemical, biophysical and biological characterization of purified VLPs is an essential component of producing VLP-based vaccines to be able to determine the functionality, potency and stability of the product [69, 112]. Examples of various analytical tools used for characterization of VLPs shown in Fig. 3.

Biochemical characterization of VLPs involves a determination of properties including primary amino acid sequence, molecular mass, isoelectric point, and purity. Mass spectrometry (MS) can be used to analyze the molecular mass of the assembled proteins, their protein sequences and their amino acid composition [112]. The molecular weights of VLPs can be measured using matrix assisted laser desorption ionization time of flight-mass spectrometry (MALDI-TOF MS) [119]. The coupling of MS with liquid chromatography provides very detailed structural information on not only amino acid sequences but also the molecular weight, disulfide bond linkages, chemical modifications, and PTMs. Thus, the liquid chromatography–mass spectrometry (LC–MS) technique is a powerful characterization tool in the production of VLP-based vaccines [120]. Sodium dodecyl sulphate polyacrylamide gel electrophoresis (SDS-PAGE) and reverse phase-high performance liquid chromatography (RP-HPLC) are the most common methods used to determine the purity, integrity and molecular weight of VLPs [112, 121]. The main disadvantage of the SDS-PAGE approach is that it is laborious and time-consuming. Due to their high sensitivity and reproducibility, RP-HPLC techniques have attractive properties for measuring the purity and the mass of VLP-based vaccines. In addition, the RP-HPLC method is useful for characterizing post-translational modifications in viral glycoproteins and can support both stages of product purity monitoring and product formulation and stability development in the VLP-based vaccine development process [122].

Biophysical parameters of VLPs such as morphology, size and polydispersity contribute to the potency and safety of VLPs vaccines. The morphology of VLPs can be detected by visualization techniques such as transmission electron microscopy (TEM), cryo-electron microscopy (Cryo-EM) and atomic force microscopy (AFM) [69, 112]. TEM is the most commonly used technique to visualize VLPs and measure particle size, but is questionable due to particle deformation in sample preparation, which can lead to particle concentration and misinterpretation of particle size. Alternative visualization techniques such as Cryo-EM and AFM are less likely to cause particle deformation due to rapid freezing and in-solution analysis during sample preparation [112, 123]. AFM is a powerful technique for measuring particle size and size distribution even in ambient conditions. In this approach, single particles can be imaged without a high cost for sample preparation [124, 125]. The measurement of VLP particle size is accomplished through asymmetric flow field-flow fractionation coupled with multiple-angle light scattering (AF4-MALS), dynamic light scattering (DLS), electrospray differential mobility analysis (ES-DMA) and high performance size exclusion chromatography (HPSEC) [69, 112]. DLS and Multi angle light scattering (MALS) are non-invasive techniques that can provide short-term measurements, although measurements of heterogeneous samples with broad size distributions tend to be underestimated due to the relative amount of smaller particles size [126]. Alternatively, ES-DMA and AF4-MALS are valuable rapid and quantitative methods to characterize multimodal VLP distributions. Both techniques can detect subtle changes in the size and other characteristic of internal packaging distribution of nucleic acids or the chimeric composition of VLP surface proteins [127]. Circular dichroism (CD), ultraviolet (UV) spectroscopy, differential scanning calorimetry (DSC) and analytical ultracentrifugation have been used to roughly characterize the biophysical properties of VLPs [119, 128]. The secondary and tertiary structures of VLPs can be measured by CD and UV spectroscopy [69]. The determination of VLP concentration measurements can be complicated due to the protein/nucleic acid content of particles. Nevertheless, UV spectroscopy has been found to be valuable for the accurate concentration measurement of VLPs [129]. Other analytical techniques for rapid and robust quantification of VLPs include methods such as HPSEC‐MALS, AF4‐MALS and nanoparticle tracking analysis (NTA). The NTA method has been demonstrated to be able to directly measure HIV-1 gag VLPs with a higher sensitivity compared to HPSEC-MALS. Although the NTA method is the most sensitive method and can be used to quantify samples with very low particle concentrations, it has many disadvantages. It is highly sensitive method and is influenced by adjustable parameters when recording and analyzing videos by the user. Furthermore, compared to AF4-MALS, NTA tends to overestimate the particles concentrations. Also, NTA analysis technique usually requires tedious sample dilution steps to prepare the sample [122, 130]. DSC and cloud point have been widely used to investigate the thermal stability and aggregation propensity of VLPs (127). The AUC technique is one of the most accurate methods for determining the size, conformation and molecular weight of VLPs [69, 112, 127].

The biological characterization of VLPs are often conducted by analyzing the binding of functional epitopes of VLPs to a panel of specific monoclonal antibodies. This binding activity is a preferred indicator of the safety and efficacy of the vaccine in vivo. Various immunoassay methods such as surface plasmon resonance (SRP), and enzyme-linked immunosorbent assay (ELISA), have been used to analyze antibody binding affinity to VLPs [69, 112, 127, 131].

## VLP-based vaccines against emerging infectious diseases

To date 110 viral proteins from 35 viral families have been shown to be capable of assembly into the VLPs [33]. From these studies, several VLP-based vaccines for human use, including Recombivax HB and Engerix-B for HBV, Gardasil, Cervarix, and Gardasil-9 for HPV and Hecolin for HEV have been licensed for clinical use. Several other VLP-based vaccines are also in various stages of design, production, and approval.

### HBV VLP-based vaccine

HBV, a virus in the family *Hepadnaviridae*, is the major causative agent of hepatitis B. HBV infection can lead to acute and chronic hepatitis and significantly increase complications and mortality [132]. Epidemiological estimates suggest that two billion people in the world have serological characteristics of hepatitis B infection, 350 million of whom have chronic hepatitis B. Vaccination is currently the most effective way to prevent HBV, and VLP-based vaccines in use are made based on the self-assembly of HBV HBsAg into VLP particles [133]. Three generations of VLP vaccines against HBV have been developed. The first generation of a HBV hematogenous VLP-based vaccine consisting of the HBsAg hepatitis B surface antigen was the Heptavax-B vaccine, known in the market as the Heptavax vaccine. The second-generation HBV vaccines, separately developed by Merck and GlaxoSmithKline, are a genetically engineered HBV VLP-based vaccine. Both companies used the Saccharomyces cerevisiae system to express the HBsAg, producing particles about 20 nm in size with a typical octagonal symmetrical structure. These vaccines are considered safer and more immunogenic than the first-generation blood-derived hepatitis B vaccine and are widely used today [134]. The third generation of the hepatitis B vaccine, Sci-B-Vac, contains three HBV antigens, including the S, Pre-S1, and Pre-S2 antigens, and is expressed in mammalian CHO cells [72, 134].

### HPV VLP-based vaccine

Persistent HPV infection is the major cause of the cervical cancer and genital warts [135]. There are currently four HPV prophylactic vaccines on the market based on self-assembled VLPs that contain only L1 protein including Gardasil (Merck), Cervarix (GSK), Gardasil-9 (Merck), and Cecolin (Innovax) [136, 137]. L1 protein is the major structural protein of the HPV that can be assembled into a VLP that is highly immunogenic and is able to elicit a type-specific immune response. Structural analysis showed that each HPV VLP contains 72 L1 pentamers [138]. Of the three vaccines, Cervarix has the lowest antigen concentration but also has a high immunogenicity and it can provide long-term protection against HPV strains 16 and 18. The formulation of Cervarix contains an AlS0_4_ adjuvant and the TLR4 MPL agonist which directly stimulates APCs [139, 140]. Due to the high cost and need for use of mammalian expression systems, prokaryotic systems are often used more for the production of HPV vaccine especially in developing countries.

Recently, a recombinant HPV type 16/18 vaccine (Solulin) was developed using the Escherichia coli expression system. This system has shown acceptable safety and performance in phase 3 clinical trials [140]. It has been suggested that in future developments VLPs can be used to express multiple antigens to protect against different strains of the same virus through chimeric design [135].

## HEV VLP-based vaccine

Hecolin, the first VLP-based vaccine for HEV launched and produced in China, has shown significant benefits in preventing HEV infection [30]. HEV causes intestinal hepatitis that occurs throughout the world and may also cause severe sporadic and epidemic acute hepatitis [141]. The length of HEV RNA genome is 7.2 kb, and contains three open reading frames, of which just ORF2 encodes a structural protein, PORF2 [142]. This structural protein contains 660 amino acids with the nuclear localization signal at the C-terminal domain (amino acid 458–607). The Hecolin VLP is a 20 to 30 nm in size and is generated from a shortened version of pORF2 (amino acid 368–606) called p239 [143]. Clinical trials have shown that Hecolin can induce high titers of HEV antibodies to provide protection from infection. It has been able to reduce hepatitis E infection by up to 93% in 4.5 years [144]. This is currently the only vaccine that can effectively prevent hepatitis E infection. Since Hecolin is produced using the *E. coli* expression system, vaccine production costs is greatly reduced and it can therefore be useful for vaccination in developing countries. In addition to Hecolin, two other HEV vaccines based on amino acids 112–607 and amino acid s439-617, p179 of pORF2 have been entered into clinical trials [143].

### Influenza virus A VLP-based vaccine

The B/IC expression system has been using to generate influenza VLPs. Sf9 cells were infected using three different baculoviruses each encoding one of the genes of the influenza hemagglutinin (HA), neuraminidase (NA), and matrix (M1) proteins. The HA and NA glycoproteins are the major antigens of the virus. Simultaneous expression of these three proteins leads to the formation of a VLP, which can be harvested from the culture supernatant. These VLPs produce a broader immune response compared to an inactivated virus or recombinant hemagglutinin protein alone. Transgenic plant technology has also been using to produce influenza VLPs that have shown promising results in the preclinical stages [145, 146].

### HIV VLP-based vaccine

HIV VLPs have been generated using various expression platforms. HIV VLPs composed of p17 and p24 structural proteins and produced by *S. cerevisiae* have now reached the clinical trials [31, 147]. Several mammalian cell lines have been used to produce HIV VLPs based on the Gag and/or envelope (env) glycoproteins proteins using transient transfection or stably transfected cell lines. Baculovirus systems and insect cells stably expressing the HIV proteins have also been used to produce gag-env VLPs [148].

### Human parvovirus VLP-based vaccine

Human parvovirus has two main structural proteins, VP1 and VP2. Human parvovirus B19 (HPVB19) VLPs composed of VP1 and VP2 proteins have reached clinical trials. These have been produced in the B/IC system in which sf9 cells are infected with two baculoviruses, leading to the production and self-assembly of immunogenic VLPs [149].

### Norovirus VLP-based vaccine

Norovirus (NV) encodes a large protein that is broken into structural proteins VP1 and VP2 and regulatory NS1/2 to NS7 proteins. The NV VLP form of the NV VLP-based vaccine used in clinical trials is composed of the VP1 capsid protein that is expressed in the B/IC system (Sf-9) has been promisingly evaluated in clinical trials. VP1-based NV VLPs have also been produced using transgenic plants and these have also undergone early clinical trials [150].

### Arenavirus VLP-based vaccine

Lassa fever virus (LASV) is a rodent-borne arenavirus that causes severe hemorrhagic fever. The LASV genome consists of two fragments of RNA (S and N). The S fragment encodes the virus nucleocapsid protein and the precursor glycoprotein (GPC). The L fragment encodes the viral polymerase (L) and the zinc finger matrix protein (Z). The major immunogenic targets of the virus are the GP1 and GP2 glycoproteins that are produced by post-translational cleavage of GPC. GP1 functions as a receptor binding protein, while GP2 is a transmembrane protein [151]. LASV VLPs can be produced using a mammalian cell line expressing the GP1 and GP2, NP and Z proteins. Mice vaccinated with a LASV VLP showed significant IgG responses to the viral proteins and serum of patients with LASV reacted to VLPs demonstrating that they are also recognised by the human immune system [152].

### Bunyavirus VLP- based vaccine

The family *Bunyaviridae* includes 5 genera including Orthobunyavirus, Phlebovirus, Nairovirus, Hantavirus that infect animals and humans and Tospovirus that infect plants. The *Bunyaviridae* genome consists of three RNA-negative segments including the large segment (L), the middle segment (M), and the small segment (S). Glycoproteins within the lipid envelope of the virion usually consist of two heterodimers, GN and GC that interact to form surface spike structures. A number of Bunyavirus VLP vaccines have been developed. Hantavirus VLPs can be obtained by expressing the virus *Gn*, *Gc* and *NP* genes in CHO cells. Animal experiments have shown that Hantavirus VLPs increase CD8^+^ T cell activity and induce antibody responses comparable to those seen with inactivated vaccines [106]. Zhou e al. developed a VLP vaccine candidate based on expression of the Bunyavirus Crimean-Congo hemorrhagic fever virus NP protein in a baculovirus VLP expression system [153].

### Filovirus VLP-based vaccine

The filoviruses VP40 matrix protein plays a critical role in the structure and assembly of these highly infectious and dangerous viruses. However, expression of VP40 alone results in poor VLP production [154]. An Ebola virus (EBOV) VLP vaccine candidate has been generated by expression of the EBOV VP40 and the virus envelop glycoprotein in 293T cells. The VLPs that arose were shown to be morphologically similar to wild-type virus particles. EBOV VLPs were highly immunogenic in in vitro and in vivo studies and they effectively induced the maturation, activation, and secretion of cytokines and chemokines. Mice vaccinated with EBOV VLPs showed B cell activation and produced high levels of EBOV-specific antibodies. The VLPs also activated CD4^+^ and CD8^+^ T cells and protected mice from deadly challenges [155].

### Paramyxovirus VLP- based vaccine

Paramyxovirus VLPs can be assembled following the simultaneous expression of viral matrix and glycoproteins. Nipah virus VLPs can be formed in HEK293T cells expressing the virus attachment glycoprotein (G), fusion (F) glycoprotein and matrix (M) protein [156]. Mice vaccinated with NiP VLPs produce specific antibodies against NiV and also produced a strong CD8^+^ T cell response. Neutralizing antibodies have also been observed in pigs vaccinated with NiP VLPs, but in these animals no CD8^+^ T cell responses were detected [157]. VLPs generated using proteins from other paramyxoviruses such as RSV, have been developed, and have shown promising results in initial pre-clinical studies [108].

### Coronaviruses VLP-based vaccine

Several animal coronaviruses have led to serious diseases in humans. These include SARS-CoV-1), Middle east respiratory syndrome-CoV (MERS-CoV) and most recently SARS-CoV-2 that has led to a global pandemic Coronavirus particles are composed of 4 structural proteins including spike (S) protein, envelope (E) protein, membrane (M) protein, and nucleoprotein (N). Experiments on SARS-CoV-1 VLPs showed that expression of SARS-CoV-1 M and E proteins in a BVES generated a smooth VLP without spikes while the simultaneous expression of M, E and S yields a structure that mimic the native SARS [51]. These remain to be assessed for protective ability.

## VLP-based vaccine as an inducer of adaptive immunity

**Fig. 4:**
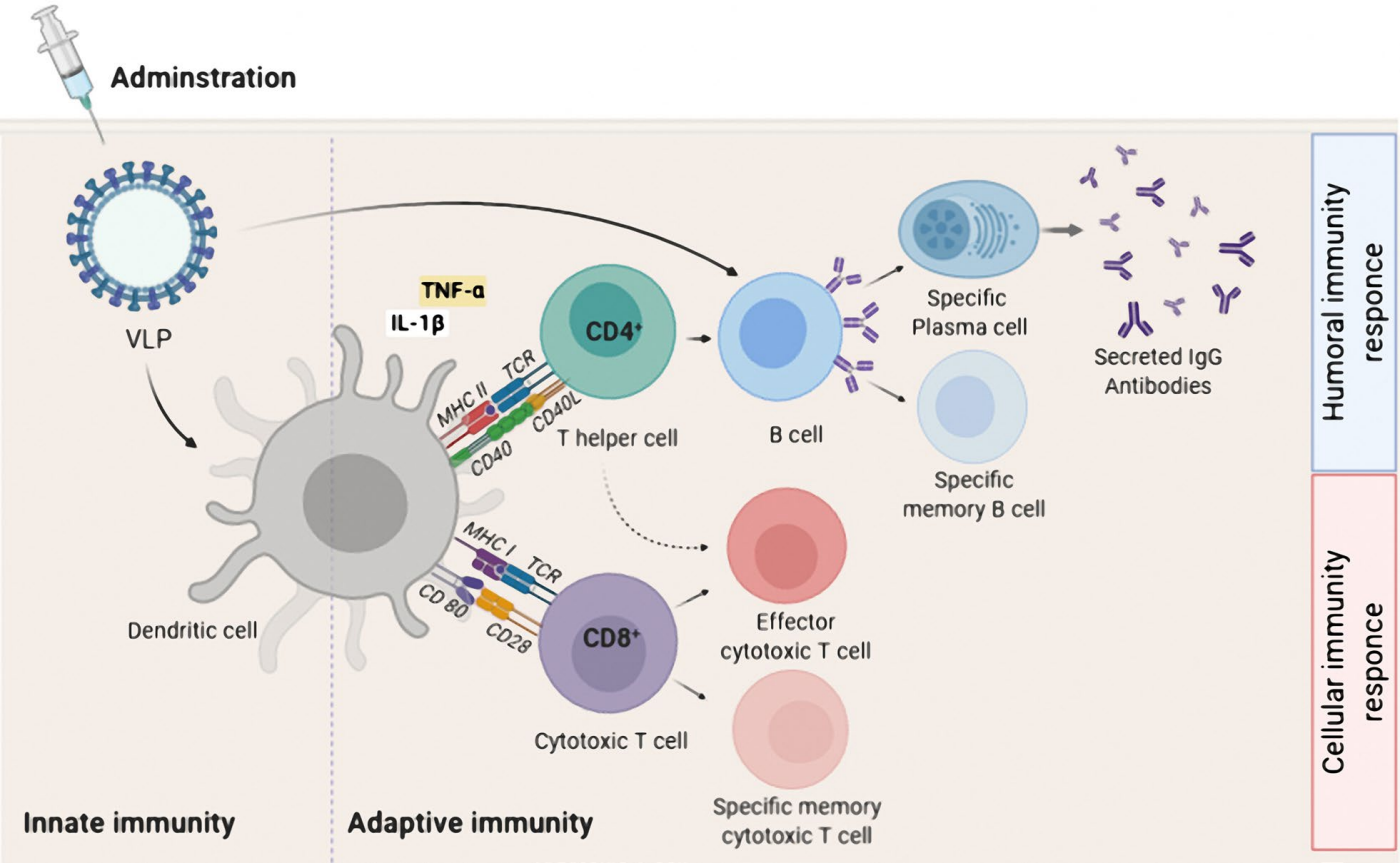
Adaptive immune activation induced by VLP-based vaccine. After administration, a VLP-based vaccine is taken up by APC such as dendritic cells. The phagocytosed VLP-based vaccine is processed and presented by both MHC-II and MHC-I for detection by CD4^+^ and CD8^+^ T cells, respectively. For induction of humoral immune responses, B cells interact with CD4^+ ^T helper cell (TH) to uptake VLP-based vaccine by B cell receptor. The interaction between CD4^+^ TH cells and B cells occurs for sufficient secretion of IgG antibodies by plasma cells as well as the generation of B memory cells. For induction of cellular immunity responses, immature CD8+ cytotoxic T lymphocytes (CTL) proliferate and differentiate into effector and specific memory CTL. Effector CD4^+^ TH cells, increase antigen presenting by APC by secreting cytokines, and also assist activated CTL

Virus capsids typically contain a repetitive protein structure that can stimulate innate immunity and induce B cells directly to produce neutralizing antibodies [158–160]. DCs are one of the most important components of APC and the bridge between innate and adaptive immunity. VLPs are usually about 10–200 nm in size and, as DCs can take up particles as small as 100–500 nm through phagocytosis and macropinocytosis, VLPs are ideally suited for acquisition before presentation of key epitopes to the immune system [161, 162]. DCs interact with VLPs through the same PRRs, such as TLRs and C-type lectin receptors (CLRs), that detect natural viruses [130, 163, 164]. VLP-based vaccines are recognized by APCs such as DCs after administration (parenteral or mucosal route) and transferred to secondary lymphoid tissues like the spleen. Recognition and uptake of the VLPs by DCs initiates the DC maturation process leading to stimulation of production of pro-inflammatory factors like TNF-α and IL-1β [165]. The pro-inflammatory factors recruit more APCs and increase the process of lysosomal proteolysis in the DCs. This leads to processing of the VLP-based vaccines into small peptides and the presentation of these peptides in the form of an MHC-peptide complex on the dendritic cell surface. Simultaneously, lymphocyte costimulatory molecules (e.g. CD80, CD86) appear on the DC surface to activate the B and T cells [166–169]. The MHC class II- peptide and costimulatory proteins activate CD4^+^ T-helper cells and T-helper cells are required for both B and T cell proliferation and differentiation processes [170–172]. In some circumstances B cell can detect the VLPs and activate humoral immunity directly and independently of the innate immunity or T helper cells (Fig. 4) [169, 173–175]. In a study by Lenz et. al. (2005), it was shown that an HPV16-based VLP is able to stimulate production of the IFN-α and IL-6 by plasmacytoid DCs (pDCs) leading to generation of antibodies [176]. In general, one of the most important benefits of VLP-based vaccines is that they are good substrates for stimulating both cellular and humoral immunity. A typical example of this is the influenza virus, which has been shown that although humoral immunity plays a key role in fighting the virus by producing neutralizing antibodies, stimulation of CD8^+^ T cells by vaccines also reduces the severity of the disease [177–179]. Similarly, HBcAg-zDIII (Zika virus envelope protein domain III) VLPs strongly stimulate both humoral and cellular immunity with only two doses of vaccine and a porcine parvovirus (PPV) VLP-based vaccine without any adjuvant strongly induced humoral and cellular immunity via both the MHC I and II class pathways [179]. Hepatitis B virus core antigen (HBcAg) VLP-based vaccine against human toxoplasmosis incorporating a B cell epitope, a CD8^+^ cell epitope, and a CD4^+^ cell epitope of *Toxoplasma gondii* stimulated both humoral and cellular immunity with greatly increased levels of IgG and IFN-ɣ, respectively [180]. The design of a VLP containing the influenza virus M2 protein showed that it could significantly increase antiviral antibody titer and provide protection against different strains of the influenza virus [177]. There are several reports of VLP-based vaccines on antibody production and cytotoxic T cell activation independent of T-helper cells. In a study using CD4^+^ T cell knockout mice animals were vaccinated with simian/human immunodeficiency virus (SHIV) VLPs or a chimeric of influenza HA/SHIV VLPs formulation. The results showed that the chimeric VLP-based system was capable of strongly stimulating humoral immunity via a T-helper cell-independent pathway. A second type of T cells, CD8^+^ cytotoxic T cells, typically detect intracellular pathogens only through the MHC class I-antigen complex [172, 181, 182]. However, VLP-based vaccines have been shown to violate this rule and can directly stimulate the MHC class I-CD8^+^ T cell pathway without the involvement of extracellular antigen. In one such study a mannosylated rabbit hemorrhagic disease virus (RHDV) VLP was presented as MHC class I-exogenous antigen and was able to directly activate cytotoxic T cells [161]. The mechanism of the cross-presentation of antigen was investigated with a p33-VLP model (epitope of lymphocytic choriomeningitis virus) showed that most of the VLP-based vaccine was taken up by CD8^−^ DCs and transferred to a secondary lymphoid organ [183]. Antigen was presented by the MHC class I complex, via two pathways one transporter associated with antigen processing [184] -dependent and the other TAP-independent with part of the antigen presented to the adaptive immune system by macrophages. A key observation was that macrophages were presented antigen, along with MHC class I proteins, only via the TAP-independent pathway. However, the processing pathways of other exogenous VLP-based vaccines to stimulate cytotoxic T cell-mediated immunity may differ. For example, a parvovirus-VLP was only taken up by CD8alpha^−^ and CD8alpha^+^ DCs via macropinocytosis and stimulated CD8^+^ T cells through the endosome-to-cytosol processing pathway [172]. There are many studies on the high immunogenicity of the VLP-based vaccines to induce humoral and cellular system, even without using other adjuvants, though the use of adjuvants generally leads to increased immunogenicity.

## VLPs for vaccination against cancer

A strong cytotoxic T lymphocyte (CTL)-mediated immune response is a major factor in eradicating tumor cells and the ability of VLPs to induce these responses by delivering antigen to the cytosol and activating the MHC class I pathway makes them excellent candidates for development of vaccines in the treatment of cancer. Several important elements of the immune system play a role in targeting cancer cells. First, DC cells must receive sufficient signals to mature and stimulate adaptive immunity. This is important to ensure that the immune system does not become tolerant to the antigen by activating regulatory T cells (Tregs) and suppressing the immune system. Second, most cancer antigens are related or identical to self-antigens. Finally, T cells must be able to overcome the immune-suppressive signals generated by tumor cells. VLPs have features that allow them to address these challenges. In this section, we consider the VLP-based vaccines that have been explored as potential anti-tumor treatments [185].

### VLP-based vaccines developed against cervical cancer

Some viral infections can lead to cancer. For example, HPV is one of these viruses, especially the two types HPV16 and HPV18, which cause 70% of cervical cancer cases [186]. The virus also causes other cancers such as anal, head, and throat and genital warts. HPV capsid consists of two important proteins called L1 (major) and L2 (minor) proteins. Two commercial vaccines, Merck’s Gardasil®, and GlaxoSmithKline’s Cervarix® have been developed to prevent HPV-related cancer. The expression systems of these two L1-based VLP vaccines are yeast and insect cells, respectively. However, these two vaccines can only prevent cancer associated with genotypes 16 and 18 (Gardasil also protects against genotypes 11 and 6, which cause benign genital warts), and do not protect against other HPV genotypes. Meanwhile these prophylactic vaccines do not cure infected people. Therefore, researchers are still trying to develop a more efficient vaccine. It has been observed that two tumor-specific antigens called E6 and E7 are expressed in all HPV infected cells and increased in cervical tumor cells [187]. E6 inactivates the P53 by binding to ubiquitin ligase E6AP and E7 degrades the phosphorylated retinoblastoma tumor suppressor pRb [188]. Thus, these two proteins repress two critical tumor suppressor proteins. HPV VLP-based vaccines are mainly L1-based because this protein can form VLP. But because the L1 is not conserved between different types of HPV, the researchers have also focused on L2 based vaccine development [189, 190]. However, the L2 challenge is its inability to form VLP [189, 191]. The concatemers consists of tandem or conserves sequences of several HPV types were designed on the MS2 bacteriophage-based VLP vaccine and the mice immunized with the construct produced high antibody titers. They also protected against different types of HPV (16, 18, 31, 33, 45 and 58) in challenge test, as the latest version of the commercial VLP-based vaccine, Gardasil9 [192]. In another study, the bacteriophage AP205 capsid-based VLP with the surface display of the HPV L2-protein and VAR2CSA placental malaria antigen was developed to protect against two diseases, cervical cancer and PM infection, simultaneously. The results showed that this system can induce humoral immunity to protect mice in challenge against both diseases [193].

### VLP-based vaccines developed against Breast cancer

Breast cancer is the most common cancer in women and affects men as well (https://www.who.int/). 20–30% of cases of invasive breast cancer is associated with overexpression of human epidermal growth factor receptor 2 (HER2) that is involved in the proliferation and inhibition of programmed cell death [194, 195]. The induction of passive immunogenicity using monoclonal antibodies has been effective in preventing metastasis and tumor growth, but this method is costly, requires multiple administrations at regular intervals for long-term protection and has undesirable side effects [196, 197]. VLP-based vaccines have been shown to address these challenges. Active vaccination with a VLP-based vaccine derived from *Acinetobacter phage* AP205 coat protein, with surface displayed HER2 protein, was evaluated in FVB mice which had been transplanted with human HER2-positive breast cancer cells. This showed that vaccination was able to inhibit tumor growth in the mice. This approach induced strong humoral immunity and also overcame immune system tolerance [198]. The product of the SLC7A11 gene, xCT, is a transmembrane protein that is overexpressed in cancer stem cells and is a target in breast cancer therapy. This protein activates Treg cells, reduces glutathione synthesis, and helps to encourage tumor invasion as Treg cells suppress the immune system to promote immune tolerance to the tumor cells [199]. A bacteriophage MS2 VLP-based vaccine that displayed the extracellular loop of xCT transporter on its surface was used to treat mice carrying metastatic breast cancer cells. The treated animals produced high levels of specific antibodies and reduced metastasis [200]. Bolli et al. developed the AX09-0M6 as a VLP-based vaccine platform by displaying of the human xCT extracellular domain (ECD6) on its surface. In vaccinated BALB/c mice the neutralizing IgG2a titer was significantly increased and the growth of breast cancer stem cells, xCT activity and pulmonary metastasis was decreased [201].

### VLP-based vaccines developed against pancreatic cancer

The cell surface glycoprotein, Mesothelin (MSLN), is overexpressed in many cancers, including pancreatic cancer, and is seen used as a potential anti-cancer drug target. This glycoprotein is involved in cell adhesion and causes cancer cell masses to attach to mesothelial cells. A VLP-based on SHIV VLPs with murine MSLN displayed on the surface of the particles was used to immunize mice harboring pancreatic tumor cells [202]. Following vaccination, tumor growth was inhibited and 60% of the treated mice survived. The VLP strongly stimulated both specific anti-tumor antibody production and CD8^+^ T cell immunity. It also prevented self-antigen suppression by inhibiting Treg cells.

An alternative therapeutic target for pancreatic cancer treatment is the transmembrane glycoprotein Trop2, which is also overexpressed in other cancers [203]. This protein has little or no expression in healthy epithelial tissue. A simian immunodeficiency virus (SIV) VLP-based vaccine presenting the Trop2 protein was examined for its efficacy against syngeneic pancreatic cancer in C57BL/6 mice [204]. Vaccinated mice showed reduced tumor growth and the VLP-based vaccine significantly activated CD4^ +^, CD8^+^ , and the natural killer cell population. Moreover, the population of Treg and myeloid‐derived suppressor cells in the tumor microenvironment was decreased. Decreased expression of immunosuppressive cytokines such as IL-10 and TGF-β confirmed that the treatment inhibited tumor-suppressing immune signals induced by the tumor cells [204].

### VLP-based vaccines developed against melanoma

Melanoma is the cause of 10% of all skin tumors and more than 90% of deaths due to skin cancer [205]. A VLP-based vaccine derived from the plant CPMV was used to assess the capacity of and empty CPMV VLP (eCPMV) to suppress tumor growth. Culture medium containing bone marrow-derived DCs (BMDCs) and primary macrophages derived from C57BL6 mice were treated with eCPMV and after 24 h an increase in the expression of some cytokines, such as IL-1β, IL-6, IL-12p40, Ccl3 (MIP1-α), and TNF-α was observed. Treatment of B16F10 lung melanoma cells with eCPMV altered tumor microenvironment immune cell organization. Tumor-infiltrating neutrophil (TIN) numbers were increased and conversely the immune-suppressing cells such as CD11b− Ly6G+ neutrophils were decreased. The mechanism of action of the vaccine was investigated using null mutant mice lacking neutrophils and cytokines IL-12 and IFNγ. In these mice, vaccination was not effective against the tumor and the vaccine did not show its protective anti-tumor effects. This strongly suggests that the beneficial effect was immune-mediated and that neutrophils and IL-12 and IFNγ played a key role in the antitumor effects [206]. The bacteriophage Qβ-based VLP system coupled with TLR9 ligands, in which the surface of each VLP was loaded with one of the Germline or mutated CTL epitopes of B16F10 were also investigated as a potential therapy with positive results. In vaccinated C57BL/6 mice with a mixture of both types of VLP the population of CD8 ^+^ T cells specific for the B16F10 murine melanoma significantly increased and tumor progression was inhibited leading to increased survival of the mice. All three types of VLPs were able to provide a degree of protection but the mixture of the two provided significant protection and prevented the progression and invasion of B16f10 cells changed the tumor microenvironment by increasing Ly6G+ granulocytic cells and decreasing Ly6C+ monocytic population [207]. These results indicate that activation of a CD8^+^ T cell mediated immune response is essential for vaccine efficacy in cancer prevention. In a study to analyze the importance of adjuvant size in stimulating T cell immunity and to evaluate the immune response in a mice model of melanoma a VLP-based platform derived from CMV and incorporating tetanus toxoid epitope TT830–843 (CMVTT-VLP) was established. The p33 peptide epitopes as a model antigen, derived from Lymphocytic choriomeningitis virus, was displayed on the particle surface and the CuMVTT-p33 VLP vaccine was formulated with micron-sized microcrystalline tyrosine (MCT) adjuvant. Comparison of the results with commercial adjuvants, Alum and B type CpGs showed that the micron-sized adjuvant stimulated CD8 + T cell immunity as much as CpG but more potent than Alum. The VLP-based vaccine showed notable antitumor effects against the B16F10 murine tumor cells [208]. VLP-based vaccines for cancer prevention have opened a new era in vaccine research, and although the results so far look promising, more research is needed to reach a definitive conclusion about the effectiveness of these vaccines. The summary of the VLP-based vaccines against different cancers are listed in Table 2.

**Table 2:** VLP-based vaccines against different cancers

VLP type	Cancer type	Antigen	Clinical phase	References
MS2	Cervical	L2	Preclinical	[192]
AP205	Cervical (and placental malaria)	HPV RG1 epitope (and VAR2CSA PM antigen)	Preclinical	[193]
AP205	Breast	HER-2	Preclinical	[190]
MS2	Breast	xCT	Preclinical	[200]
MS2	Breast	xCT	Preclinical	[201]
SHIV	Pancreatic	hMSLN	Preclinical	[202]
SIV	Pancreatic	mTrop2	Preclinical	[204]
eCPMV	Melanoma	Empty	Preclinical	[204]
Cucumber mosaic VLPs (CMV)	Melanoma	LCMV-gp33	Preclinical	[208]
Bacteriophage Qβ	Melanoma	PMEL17, MTC-1, Calpastatin, ZFP518, TRP-2, Caveolin2, Cpsf3l and Kifl8b	Preclinical	[208]

## Application of VLPs in drug delivery

Although VLPs are best known for their immunogenic properties, other capabilities of these nanoparticles have recently been considered, including their applications in drug delivery and gene therapy. In addition to carrying peptides/proteins or other active molecules displayed on the surface of the VLPs, they have the ability to entrap proteins, nucleic acids, or other small molecules. Therefore, they can be used as a means of delivering these molecules to specific cells, tissues, or organs [209].

Cells use receptor-mediated endocytosis for uptake of VLPs. During endocytosis, the plasma membrane surrounds the VLPs and buds off inside the cell as a vesicle. Then, the vesicle is separated from the membrane and enters the cytosol. The released vesicles are transported along the cytoskeleton to combine with the primary endosomes. Eventually, the endosomal vesicles separate from the primary endosome, mature as the final endosome and merging with the pre-lysosomal vesicles containing acidic hydrolase to form lysosomes. The foreign materials are broken down inside the lysosomes and made available to the cells. However, lysosomal degradation prevents proper drug delivery, so that about 40% of newly produced drugs are disapproved due to poor bioavailability. The use of drug nanocarriers is a good strategy to overcome this limitation. So far, there are several nanocarriers that have been developed using different methods. Among nanocarriers, VLPs are highly suitable for drug delivery purposes due to their ability to escape endosomes before lysosomal degradation [209]. VLPs have numerous features that make them ideal for targeted drug delivery. Delivery of materials using these NPs can provide targeted and intracellular drug delivery, increase the accumulation and bioavailability of drug in specific sites, e.g., tumor tissues, minimize the required dose of drug and improve treatment outcomes. Some VLPs show a natural tropism toward a particular tissue that is due to the virus from which they originated. For example, because HBV naturally infects the liver, HBV-derived VLPs can target liver cells. Similarly, rotaviruses show a special affinity for the intestine, so that this feature of their derived VLPs can be used for targeted delivery of the drug to the intestinal tissue [209].

More specific targeting is usually achieved by displaying receptor-binding domain on the VLP surface. Target domains can be chemically or genetically attached to the surface of VLPs, which allows the VLPs to selectively bind to cancer cells that express a specific receptor and enhance the therapeutic effects of drugs [68].

VLPs can also be used to deliver nucleic acids. For example, one study showed that systematic delivery of miR-146a, a known gene silencer, via bacteriophage MS2 -derived VLPs, is an effective treatment for reducing inflammatory cytokines in mice susceptible to systemic lupus erythematosus [68]. Examples of successful applications of VLPs for drug delivery are shown in Table 3.

**Table 3:** Application of VLPs as drug delivery

References	Application	Cargo (Drug, Nucleic acids, Proteins)	VLP
[210–212]	Tumor therapy	Bleomycin (BLM) Paclitaxel (PTX) mRNA cap analog	Adenovirus (AdV)
[213]	Tumor therapy	Doxorubicin, Cisplatin, 5-fluorouracil siRNA Ricin toxin A-chain (RTA)	Bacteriophage MS2
[214]	Tumor therapy	Doxorubicin (DOX)	Rotavirus [66]
[215]	Tumor therapy	Doxorubicin (DOX)	*Cowpea mosaic virus* (CPMV)
[216]	Tumor therapy	Doxorubicin (Dox)	*Cucumber mosaic virus* (CMV)
[217]	Tumor therapy	siRNA	Hepatitis B virus (HBV)
[218]	Tumor therapy	Methotrexate (MTX)	Polyomavirus
[219]	Antimicrobial drug	Chloramphenicol	Filamentous bacteriophages (fd or M13)
[220]	Antimicrobial drug	Azithromycin/clarithromycin	Bacteriophage Qβ

## Conclusions

With rapid advances in nanotechnology and protein engineering, interesting capabilities have been developed for the development and improvement of vaccine carriers as well as packaging and delivery tools for drugs. In recent years, viruses have been considered as not only to be the cause of disease, but also as functional NPs that are useful for a variety of applications. Self-assembled VLPs are one of these nanostructures with the ability to deliver antigens and drugs to various targets within tissues and organs. Traditional vaccines are often produced by inactivating or attenuating viral strains and genome-free VLPs offer new and safer alternatives. The variety of VLPs makes them structurally attractive and functionally diverse and VLPs can be designed to carry polyvalent antigenic structures that can also deliver antigenic compounds to specific target tissues. As well as being immunogens in their own right. VLPs have also been successfully used as adjuvants to elicit a strong immune response. By choosing appropriate types of VLPs, it is possible to stimulate both the innate and adaptive immune systems and in some cases VLP-based vaccines without any adjuvants have been shown to stimulate humoral and cellular immunity through the MHC class I and II pathway.

VLPs can be used not only as preventive vaccines by displaying the foreign antigens on their surface to stimulate the immune system and to prevent infectious diseases, but also as therapeutic vaccines to present patient’s own antigens and to help them fighting against chronic and metabolic diseases or different types of cancers. Several VLP-derived vaccines are commercially licensed or are under evaluation in clinical trials. However, more evidence is needed to fully assess the potential efficacy, side effects, challenges and benefits of VLP-based vaccines in the treatment or prevention of various types of cancers.

Technical challenges such as getting molecules to display on the particle surface properly remain but the continuing study of these structures is providing a considerable body of information to successfully address these problems. A potential solution to overcome this problem, which has been developed in our lab, is the insertion of a sortase recognition motif (LPXTG) into different parts of VLP that are likely to be exposed on the particle surface. In this way, it is possible to retain the integrity of the VLP and make it possible to conjugate any protein on the VLP surface [221–223]. Other application for changing the external surface of VLPs is delivering the drug to a specific cell or tissue to treat a specific disease. The surface of the VLP can be changed so that the desired VLP enters the specific tissue. For this purpose, molecules that must be delivered on the surface, are fused to this set to deliberately deliver this complex to a specific target [224]. Although the use of VLP-based vaccines has had significant success in preventing disease, there are still problems in this area, and more time and research is needed to reach the ideal state to address these challenges.

## Data Availability

Not applicable.

## Abbreviations

VLPs: Virus-like particles
HIV: Human immunodeficiency virus
HBV: Hepatitis B virus
HCV: Hepatitis C virus
PASP: Pathogen-associated structural patterns
EPR: Enhanced permeability and retention
HPV: Human papillomavirus
eVLPs: Enveloped VLPs
DENV: Dengue viruses
JEV: Japanese encephalitis virus
LSP: Large-scale production and purification
BVES: Baculovirus expression system
PTM: Post-translational modifications
HEV: Hepatitis E virus
WNV: West Nile virus
FMS: Foot-and-mouth disease
CCMV: Cowpea chlorotic mottle virus
CHIK: Chikungunya
SARS: Severe acute respiratory syndrome
RVFV: Rift Valley fever virus
TMV: Tobacco mosaic virus
CMV: Cucumber mosaic virus
AIMV: Alfalfa mosaic virus
CPMV: Cowpea mosaic virus
PapMV: Papaya mosaic virus
PVX: Potato X virus
PMMoV: Pepper mild mottle virus
APCs: Antigen-presenting complexes
DCs: Dendritic cells
NV: Norwalk virus
NVCP: Norwalk virus capsid
CPMV-HT: Cowpea mosaic virus-Hyper Translatable
AHS: African horse sickness
CHO: Chinese hamster ovary
HBsAg: Hepatitis B virus surface antigens
BHK-21: Baby hamster kidney-21
HEK293: Human embryonic kidney 293
ELL-0: East lansing line-0
NDV: Newcastle disease virus
RSV: Respiratory syncytial virus
CFPS: Cell-free protein synthesis
UAAs: Unnatural amino acids
TFF: Tangential flow filtration
UF/DF: Ultrafiltration/Diafiltration
IEC: Ion-exchange chromatography
HIC: Hydrophobic interaction chromatography
SEC: Size exclusion chromatography
PRRs: Pattern recognition receptors
IL12: Interleukin 12
MPLA: Monophosphoryl lipid A
ODN: Oligonucleotide
TLRs: Toll like receptors
CT: Cholera toxin
LT: Heat-labile toxin
MS: Mass spectrometry
SDS-PAGE: Sodium dodecyl sulphate polyacrylamide gel electrophoresis
RPHPLC: Reverse phase- high performance liquid chromatography
TEM: Transmission electron microscopy
Cryo-EM: Cryo-electron microscopy
AFM: Atomic force microscopy
AF4-MALS: Asymmetric flow field-flow fractionation-Multiangle light scattering
DLS: Dynamic light scattering
ES-DMA: Electrospray differential mobility analysis
HPSEC: High performance size exclusion chromatography
CD: Circular dichroism
DSC: Differential scanning calorimetry
SRP: Surface plasmon resonance
ELISA: Enzyme-linked immunosorbent assay
HPVB19: Human parvovirus B19
LASV: Lassa fever virus
EBOV: Ebola virus
MERS-CoV: Middle east respiratory syndrome-CoV
CLRs: C-type lectin receptors
HBcAg: Hepatitis B virus core antigen
RHDV: Rabbit hemorrhagic disease virus
SHIV: Simian HIV
CTL: Cytotoxic T lymphocyte
PM: Placental malaria
HER2: Human epidermal growth factor receptor 2
SIV: Simian immunodeficiency virus
BMDCs: Bone marrow-derived dendritic cells
TIN: Tumor-infiltrating neutrophil
MCT: Microcrystalline tyrosine
